# The Nigerian Bioinformatics and Genomics Network (NBGN): a collaborative platform to advance bioinformatics and genomics in Nigeria

**DOI:** 10.1017/gheg.2020.3

**Published:** 2020-07-15

**Authors:** Segun Fatumo, ThankGod E. Ebenezer, Chinwe Ekenna, Itunuoluwa Isewon, Umar Ahmad, Charles Adetunji, Elijah Kolawole Oladipo, Marion Adebiyi, Ezekiel Adebiyi, Oyekanmi Nashiru, The Nigerian Bioinformatics and Genomics Network

**Affiliations:** 1 London School of Hygiene and Tropical Medicine, London, UK; 2 Uganda Medical Informatics Centre, MRC/UVRI and LSHTM Uganda Research Unit, Entebbe, Uganda; 3 H3 Africa Bioinformatics Network (H3 ABioNet) Node, CGRI/NABDA, Abuja, Nigeria; 4 Organisms and Ecosystems, Earlham Institute, Norwich Research Park Innovation Centre, Norwich NR4 7UZ, UK; 5 University at Albany, New York, USA; 6 Department of Computer and Information Sciences, Covenant University, Ota, Nigeria; 7 Medical Genetics Laboratory, Genetics and Regenerative Medicine Research Centre, Faculty of Medicine and Health Sciences, Universiti Putra, Selangor, Malaysia; 8 Department of Anatomy, Faculty of Medicine, Bauchi State University, Gadau, Nigeria; 9 Edo University Iyamho, Iyamho, Nigeria; 10 Department of Microbiology, Laboratory of Molecular Biology, Immunology and Bioinformatics, Adeleke University, Ede, Osun State, Nigeria; 11 Department of Computer Science, Landmark University, Omu-Aran, Nigeria

**Keywords:** Africa, bioinformatics, genomics, NBGN Nigeria

## Abstract

Africa plays a central importance role in the human origins, and disease susceptibility, agriculture and biodiversity conservation. Nigeria as the most populous and most diverse country in Africa, owing to its 250 ethnic groups and over 500 different native languages is imperative to any global genomic initiative. The newly inaugurated Nigerian Bioinformatics and Genomics Network (NBGN) becomes necessary to facilitate research collaborative activities and foster opportunities for skills’ development amongst Nigerian bioinformatics and genomics investigators. NBGN aims to advance and sustain the fields of genomics and bioinformatics in Nigeria by serving as a vehicle to foster collaboration, provision of new opportunities for interactions between various interdisciplinary subfields of genomics, computational biology and bioinformatics as this will provide opportunities for early career researchers. To provide the foundation for sustainable collaborations, the network organises conferences, workshops, trainings and create opportunities for collaborative research studies and internships, recognise excellence, openly share information and create opportunities for more Nigerians to develop the necessary skills to exceed in genomics and bioinformatics. NBGN currently has attracted more than 650 members around the world. Research collaborations between Nigeria, Africa and the West will grow and all stakeholders, including funding partners, African scientists, researchers across the globe, physicians and patients will be the eventual winners. The exponential membership growth and diversity of research interests of NBGN just within weeks of its establishment and the unanticipated attendance of its activities suggest the significant importance of the network to bioinformatics and genomics research in Nigeria.

## Introduction

Over the past two decades, there have been major advances in genomic studies globally with the most significant efforts aimed at investigating the genetic basis of human diseases [1–5]. Many of these studies have emphasised the central importance of Africa to the human origins, disease susceptibility, agriculture, and biodiversity conservation [6]. Nigeria is the most populous and most diverse country in Africa and thus plays a prominent role in population genetics in Africa and the world at large, owing to its 250 ethnic groups and over 500 different native languages, which are characterised by greater levels of genetic diversity and broad population substructure. Since the inception of the significant initiative such as Human Heredity and Health in Africa (H3Africa) consortium (http://h3africa.org) [7], the amount of genomic data generated in Africa has surged. Given the leading roles, many Nigerians play in emerging genomic projects and low footprints of Nigerian trained Genomicists and Bioinformaticians [8,9]. Therefore, the newly inaugurated Nigerian Bioinformatics and Genomics Network (NBGN) becomes necessary to facilitate collaborative activities amongst Nigerian bioinformatics and genomics investigators. NBGN aims to advance and sustain the fields of genomics and bioinformatics in Nigeria by serving as a vehicle to foster collaboration, provision of new opportunities for interactions between various interdisciplinary subfields of genomics, computational biology and bioinformatics, provide opportunities for the early career researchers.

**Fig. 1.**
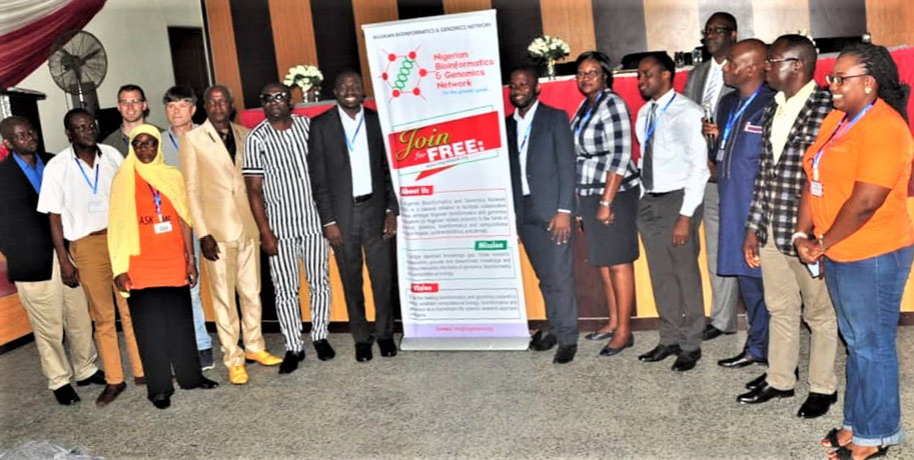
NBGN inauguration ceremony. Picture of NBGN pioneer executives, keynote speakers, invited dignitaries, and some members of NBGN taken during the inauguration ceremony on Wednesday 26 June 2019.

The NBGN was inaugurated on 26 June 2019 at the Nigerian Institute of Medical Research (NIMR), Lagos, Nigeria (Fig. 1) during the Nigerian's premier bioinformatics conference. The inauguration featured presentations by current executives about the activities of the network since inception and goodwill messages by invited dignitaries and leaders of affiliate societies such as Genetics Society of Nigeria (GSN), Biotechnology Society of Nigeria (BSN), Nigerian Society of Bioinformatics and Computational Biology (NiSBCB), Nigerian Bioinformatics and Education Research Network (NBREN) and Nigerian Society for Human Genetics (NiSHG). Two keynote presentations were given by Prof. Oyekanmi Nashiru and Prof. Ezekiel Adebiyi. One of the highlights of the inauguration ceremony was a special recognition award presented to Prof. Oyekanmi Nashiru and Prof. Ezekiel Adebiyi by the NBGN executives in recognition of their excellent leadership and pioneer effort in driving bioinformatics research and training in Nigeria amidst minimal infrastructure and several other challenges.

Bioinformatics and genomics are relatively new scientific disciplines, with surge of interest only in the last two decades. In Nigeria, there has also been considerable engagements by investigators, and the role of Nigeria in these initiatives has previously been reported [8,10]. A metrics for the measurement of this surge of interest could be seen in the increased amount of publications affiliated with Nigerian institutions over the last 5 years (online Supplementary Fig. S1). For instance, a search on PubMed using the same search query between 2014 and 2019 retrieved a total 368 publications with 2014 having the least number of publications of 34 (online Supplementary Fig. S1). The results are indeed expected as a number of research and capacity building interventions and funding have happened over the past 5 years with an increased research, education and collaborations within the Nigerian bioinformatics and genomics community. For instance, several bioinformatics and genomics research projects have recently been launched such as the Centre for Genomics Research and Innovation (CGRI) at the National Biotechnology Development Agency (NBDA), Genomic Characterization and Surveillance of Microbial Threats in West Africa at Redeemer's University, Ede, Nigeria, the African Collaborative Centre for Microbiome and Genomics Research (ACCME), and the Cassava and Yam projects at the International Institute of Tropical Agriculture (IITA), Ibadan, Nigeria. Aside from the heavily funded bioinformatics and genomics projects, several other bioinformatics and genomics communities have emerged to build capacity at their institutional and organisational levels at the grassroot levels. For instance, these groups include, Nnamdi Azikiwe University Bioinformatics and Genomics Consortium (NBGC), the Bioinformatics Training at Adeleke University, Ede, as well as the Molecular Docking and Training workshops at Bayero University, Kano. This list of bioinformatics and genomics funded projects and communities in Nigeria does not aim to be exhaustive, but to provide an initial insight into the state of the national bioinformatics and genomics community. Therefore, the NBGN aims to build connections and promote collaboration between these increasing activities in Nigeria that will help in accelerating research and innovation in the field of genetics, genomics, bioinformatics, and computational biology within Nigeria, continental Africa, and the global regions.

## Organisation, leadership, and memberships

NBGN is currently made up of the identified pioneering leadership, identified society representative slots (e.g. Nigeria Society of Computational Biology and Bioinformatics, Biotechnology Society of Nigeria, Genetic Society of Nigeria, Nigeria Society for Human Genetics, etc.) – all of these make up the governing council which currently being driven by the pioneering leadership. All positions are currently vacant apart from the pioneering leadership which is made up of the founder and President (Segun Fatumo), Vice President – Communications & Operations (ThankGod Ebenezer), and Vice President – Finance & Logistics (Chinwe Ekenna) (http://www.nbgnetwork.org). There are provisions for a Network Advisory Board (NAB) which will be made up of senior scientists with sufficient scientific research, policy, and leadership experience.

**Fig. 2.**
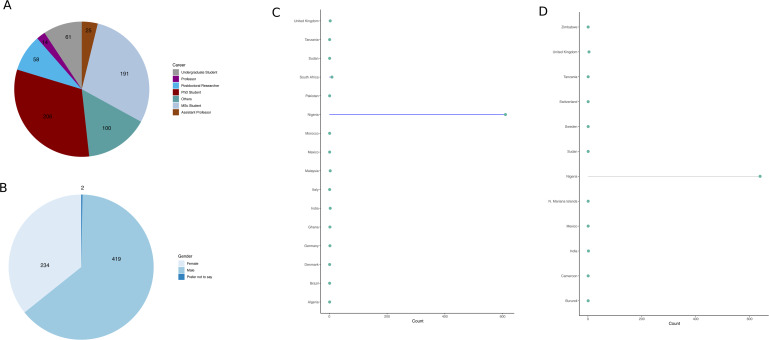
Membership distribution of NBGN members. Panel A depicts NBGN membership across career stages. Legends correspond to undergraduates, Ph.D. students, M.Sc. students, professor, postdoctoral researcher, assistant professor, and others, respectively. Ph.D. students recorded the highest number of memberships. Panel B depicts NBGN membership across gender. Legends correspond to male, female, and prefer not to say. There are fewer females than males. Panel C depicts membership NBGN distributions across countries of affiliations. Coloured node suggests intersect between number of members and respective countries of affiliations. There are currently 16 countries of affiliations within NBGN. The *y*-axis corresponds to countries of affiliations; the *x*-axis corresponds to the number of members. Panel D depicts membership NBGN distributions across countries of nationalities. Coloured node suggests intersect between number of members and respective countries of nationalities. There are currently 12 groups of nationalities within NBGN. The *y*-axis corresponds to countries of nationalities; the *x*-axis corresponds to the number of members. Note that figures were plotted using ggplot2 in R.

NBGN engages the public through social media such as Facebook, Telegram, Instagram, and LinkedIn as well as through membership subscription-based system. There are two membership streams: individual and partners. All membership streams are free until June 2020 with up to 655 current members (individual membership stream) with Ph.D. students recording the highest number of memberships (Fig. 2A). Membership also cuts across up to 16 countries of affiliations (Fig. 2C) and up to 12 nationalities (Fig. 2D).

## The opportunities proffered by the NBGN

Advances in genome technology has led to the establishment of various societies, organisations, and networks that sought to share ideas, face challenges, and prosper solutions to human problems through bioinformatics and computational biology applications. In any network or organisational space, there is usually a provision of shared opportunities for its members community and immediate society to bring about benefit. Bioinformatics networks and societies play a vital role in the promotion and capacity building of many scientists across the world, including Nigerian state, through collaborations and networking. By fostering this networking, a number of many human problems that include health-related issues, agriculture and environment were remarkably addressed [11–13]. NBGN therefore aims to provide several opportunities for scientists, early career researchers as well as students with passionate interest in bioinformatics, genomics, and computational biology-related studies in Nigeria. Engaging and participating in NBGN activities offer networking and collaborations with other local scientists who might be working in the field of your research interest in Nigeria, Africa, and the rest of the world.

The recent first Nigerian bioinformatics conference (FNBC) organised by NBGN has demonstrated how the network helped in bridging the knowledge gap, fostering research collaborations, and providing peer support within the field of bioinformatics and computational biology [14]. Bioinformatics and genomic trainings in the form of workshops, seminars, short courses, and symposia are offered by NBGN to support and provide basic bioinformatics skills to Nigerian scientists working in the field of biomedical and life sciences as well as clinical settings in our local institutions, research centres, and hospitals. The training will provide strong foundation and enhance the knowledge of our local scientists and researchers on bioinformatics and computational genomics with the hope to step down the training to their various institutions and organisations. NBGN hopes to ensure that an integral part of the undergraduate programmes in life sciences, biomedical, medical, and biological sciences curricula covered some bioinformatics courses and is looking forward to collaborate with the local institutions to develop a specialised M.Sc. and Ph.D. degree programmes in bioinformatics.

## Discussion and conclusion

The current state of genomic and bioinformatics in Nigeria in terms of resources, expertise, and infrastructure is still far from meeting its research needs, diversity and the Nigeria population. The NBGN is geared towards bridging knowledge gap, fostering research collaboration, and provide opportunities to strengthen genomics and bioinformatics in Nigeria.

NBGN is at a take-off point where the coalition of diverse researchers has the potential to put Nigeria on the map with regards to quality research undertakings in Africa and the rest of the world. The establishment of NBGN pushes the frontier on what can be achieved with a well-designed and organised network. The recently concluded First Nigerian Bioinformatics Conference (FNBC) [14] was a success and serves as a proof of concept to the rising need for a strong coalition and collaboration framework in Nigeria with a potential to extend beyond its frontiers. The rise on the membership counts for NBGN pinpoints to the human capacity available, which we hope the framework will harness.

NBGN organised two well attended pre-conference workshops with trainers from the European Bioinformatics Institute and University of Cape Town [14]. The FNBC organised by NBGN recorded 195 online registered participants, and 186 actual participants; comprising eight keynote speakers, six invited speakers, 25 oral presenters, 83 poster presenters, and up to 73 non-presenting participants including 16 international attendees [14]. NBGN having started on a strong footing, has continue to be driven by the pioneering leadership as well as some societal representative drawn from each of the aforementioned scientific and professional societies.

Finally, looking into the near future the entire leadership of the network will be made up of earnest researchers willing to open doors to international collaborations, attract quality opportunity that will potentially lead to the best practice research within the NBGN community.

## Supplementary material

For supplementary material accompanying this paper visit http://dx.doi.org/10.1017/gheg.2020.3. click here to view supplementary material
