# Supplementary material for: The Nigerian Bioinformatics and Genomics Network (NBGN): a collaborative platform to advance bioinformatics and genomics in Nigeria
Source: Glob Health Epidemiol Genom. 2020 Jul 15;5:e3. doi: 10.1017/gheg.2020.3 (PMC7372179; doi:10.1017/gheg.2020.3)
Supplement: Supplementary file 1 [file S2054420020000032sup001.pdf]

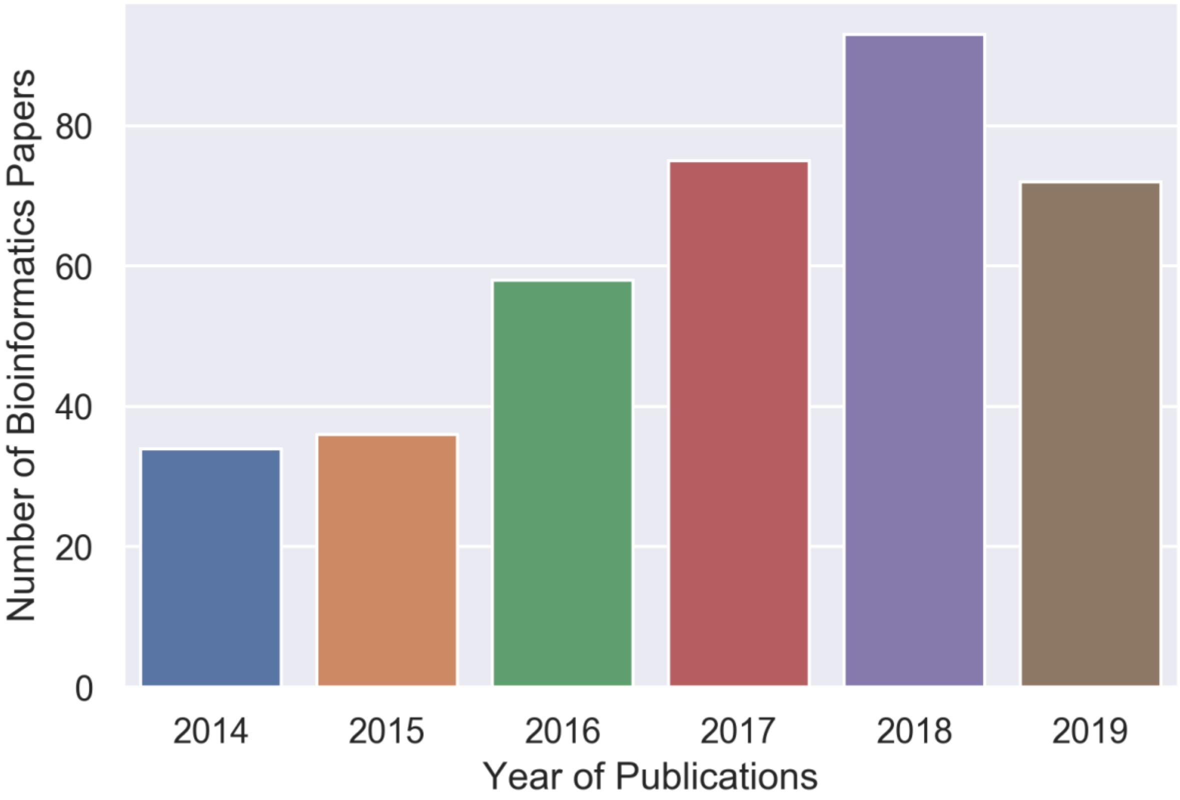

Supplementary Figure S1 | Bioinformatics related publications by Nigerian Scientists from 2014 to 2019. Publications were retrieved from PubMed search using the following search terms; ‘‘bioinformatics Nigeria’’, ‘‘*in silico* Nigeria’’, ‘‘computational biology Nigeria’’ and ‘‘next generation sequencing Nigeria’’ from 2014 to 2019. Note that the figure was plotted using seaborn library in Python.
